# Autologous Fat Plus Platelet-Rich Plasma versus Autologous Fat Alone on Sulcus Vocalis

**DOI:** 10.3390/jcm11030725

**Published:** 2022-01-29

**Authors:** Yung-An Tsou, Vincent Hui-Chi Tien, Sheng-Hwa Chen, Liang-Chun Shih, Tzu-Chieh Lin, Chien-Jen Chiu, Wen-Dien Chang

**Affiliations:** 1 Department of Otolaryngology-Head and Neck Surgery, China Medical University Hospital, Taichung 40402, Taiwan; d22052121@gmail.com (Y.-A.T.); vincenttien0623@asia.edu.tw (V.H.-C.T.); d14365@mail.cmuh.org.tw (L.-C.S.); drofarmyhospital@yahoo.com.tw (T.-C.L.); blueness1103@hotmail.com (C.-J.C.); 2 School of Medicine, China Medical University, Taichung 40402, Taiwan; 3 Department of Otolaryngology Head and Neck Surgery, Asia University Hospital, Taichung 40402, Taiwan; 4 Department of Audiology and Speech-Language Pathology, Asia University, Taichung 41354, Taiwan; shchen@asia.edu.tw; 5 Graduate Institute of Biomedical Sciences, China Medical University, Taichung 40402, Taiwan; 6 Department of Sport Performance, National Taiwan University of Sport, Taichung 404401, Taiwan

**Keywords:** sulcus vocalis, lamina propria, platelet-rich plasma, autologous fat

## Abstract

Sulcus vocalis is a frequent cause of glottic insufficiency that leads to incomplete vocal fold closure during phonation. Type II sulcus vocalis is defined as a partial defect of the lamina propria (LP). Treatment with fillers, such as fat or hyaluronic acid (HA), in the vocal folds is widely used, but the duration of effect is variable. Platelet-rich plasma (PRP) can enhance the survival of autologous fat in fat grafting, and also is used to treat sulcus vocalis. This study aimed to compare the effectiveness of autologous fat graft versus fat graft plus PRP to treat type II sulcus vocalis. Thirty-four patients with a voice handicap index (VHI) ≥ 11 were randomized to two groups, which received LP injections of fat graft (*n* = 17) or fat graft plus PRP (*n* = 17). At 1 month and 6 months after injection, the VHI decreased significantly in both groups. The fat plus PRP group had better Jitter, Shimmer, and noise to harmonic ratio (NHR) in 1 month and 6 months after surgery. The fat plus PRP group resulted in lower VHI scores one month after surgery, and stroboscopy revealed sustained smaller gaps after six months. These results indicate that a combination of fat graft plus PRP is safe and effective for treating sulcus vocalis type II and associated vocal atrophy.

## 1. Introduction

Vocal lamina propria defects are the leading cause of sulcus vocalis and are defined as type I, type II, and type III sulcus vocalis based on different degrees of injury of the lamina propria [1]. Injection laryngoplasty is the first-line treatment for glottic insufficiency, and early injection laryngoplasty resulted in better voice outcomes and decreased the need for permanent medialization thyroplasty [2]. Apart from vocal palsy, the cause of glottic insufficiency is multifactorial, including vocal paralysis, vocal tumor, recurrent laryngeal neuropathy, myopathy, and vocal sulcus [3], which is considered a defect of Reinke’s space. The three types of sulcus vocalis are defined by the degree of epithelial invagination into the superficial lamina propria (SLP). Type I is considered to limit the SLP and have no functional impact. Type II has a greater atrophic mucosa than type I, with partial loss of the SLP. Type III has a large degree of deficiency and near-total loss of the SLP with the epithelium nearly contacting the vocal ligament [4]. The etiology of the vocal sulcus is considered congenital in origin due to the abnormal development of the vocal fold during embryogenesis. Acquired vocal sulcus may be due to vocal cyst rupture and formation of a vocal mucosa bridge, overuse of the vocal folds (phonotrauma), or vocal fold inflammation [5].

Methods for treating vocal atrophy with sulcus vocalis include fat injection laryngoplasty, hyaluronic acid (HA) injection laryngoplasty, and bilateral type I thyroplasty [6]. Injection laryngoplasty is an important treatment method for vocal atrophy with vocal sulcus. The injection of fillers, such as fat and HA, is commonly used to treat glottal insufficiency [7]. Regardless of the treatment method, the restoration of lamina propria (LP) in the vocal fold mucosa is difficult. This is primarily because the extracellular matrix (ECM) components of the LP in the vocal folds remain unchanged (scarring, collagen deposition) even after filler treatment. In addition, possible absorption of injection content (e.g., HA, autologous fat) can lead to the recurrence of poor voice quality with time [7]. Of the potential materials that can be injected, autologous fat injection is currently considered medialization injection laryngoplasty as well as regenerative injection laryngoplasty [8]. Longer stable voice outcomes are achieved by autologous fat injection laryngoplasty; however, reabsorption of the fat is inevitable in certain conditions. As such, stem cell therapy or the injection of a fat and platelet-rich plasma (PRP) mixture has been considered to gain a prolonged and more stable treatment outcome for glottal insufficiency [9].

PRP is produced by centrifugation of blood and is rich in platelets and growth factors that promote tissue regeneration and even muscle regeneration [10]. For vocal fold injection, PRP is prepared from the patient’s blood. However, PRP is readily absorbed, and thus the effect for sulcus vocalis is only short term [9]. An ideal injection material should have a long-term effect and be simple to prepare. A blood centrifuge time of 20 min results in a high concentration of platelets and a high concentration of growth factors. It removes most of the white blood cells (WBCs) and related cytokines that might cause inflammation. Therefore, we measured the outcomes to see whether the injection of a mixture of the highly concentrated PRP and autologous fat could provide a more prolonged treatment effect for sulcus vocalis. Thus, the purpose of this study was to compare the effect of an injection of autologous fat alone with that of a mixture of PRP plus autologous fat for the treatment of type II vocal sulcus.

## 2. Materials and Methods

**Figure 1:**
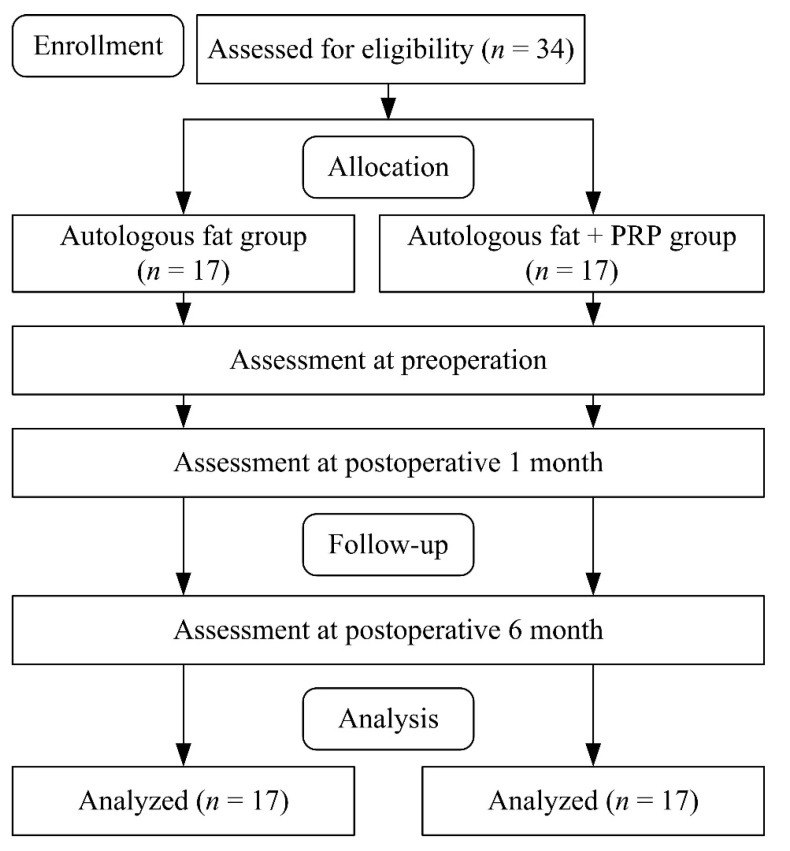
The study procedure.

Patients from the otolaryngology (head and neck surgery) department of China Medical University hospital with type II sulcus vocalis were recruited for this study. The Institutional Review Board approved this study at CMU Hospital. A flow diagram of patient selection and study procedures is shown in Figure 1.

Patients with vocal atrophy and sulcus vocalis type II diagnosed by direct suspension laryngomicrosurgery between January 2020 and March 2021 were eligible for inclusion. The inclusion and exclusion criteria in this study are presented in Table 1. All patients underwent preoperative stroboscopy, and two senior laryngologists and a speech pathologist confirmed type II sulcus vocalis by stroboscopy. The diagnosis of sulcus was also confirmed during microlaryngoscopy injection laryngoplasty. During the injection laryngoplasty, a senior laryngologist (with 15 years of laryngeal surgery experience) directly palpated the vocal fold epithelium to see the extent and severity of the sulcus vocalis. Patients were randomly divided into a group treated by autologous fat injection laryngoplasty alone (autologous fat group). A group was treated by injecting a mixture of autologous plus PRP (autologous fat plus PRP group). All of the participants were blinded to which group they were in. A videostroboscopic examination and acoustic analysis took place and grade, roughness, breathiness, asthenia, strain scale (GRBAS), and voice handicap index-10 (VHI-10) were assessed by the same otolaryngologist before surgery and at 1 month and 6 months after surgery. All patients also received a voice recording and a multidimensional voice program (MDVP) voice analysis before surgery and at 1 month and 6 months after surgery. The procedures of surgery and injection preparation were conducted by two laryngologists, and all assessments were measured by one experienced speech pathologist. Another researcher collected and analyzed the data. All patients were blinded to participant allocation and intervention, but all the researchers were not blinded in this study.

### 2.1. Procedure

**Figure 2:**
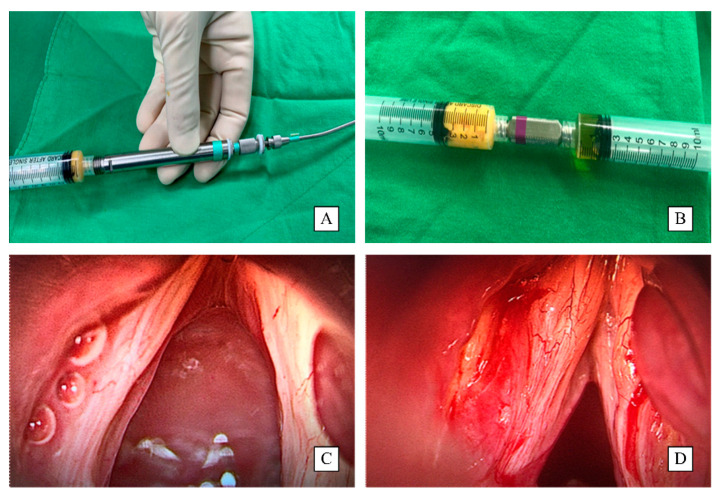
Preparation of PRP and autologous fat mixture for injection laryngoplasty: (**A**) the fat was harvested by an 18-gauge needle fat harvesting syringe, (**B**) PRP and autologous fat mixture, and (**C**,**D**) the change of injection site before (**C**) and after (**D**) the operation with PRP and autologous fat mixture.

All patients underwent injection laryngoplasty under general anesthesia performed by a senior laryngologist with the assistance of a laryngology fellow. The laryngology fellow completed the process of fat harvesting and injection preparation. The fat was harvested from lower abdominal subcutis fat with an 18-gauge needle with a fat harvesting syringe, and 30–40 mL of subcutaneous adipose soft tissue was obtained (Figure 2). The harvested fat was centrifuged at 3000 rpm for 5 min, and then the blood at the bottom of the tube and liquid oil at the top of the tube were removed after 5 min at room temperature. The mid-layer fat was harvested and washed with normal saline at room temperature to remove any blood such that only emulsified fat remained [11]. PRP was collected by centrifuging 10 mL of whole blood for 20 min to elude the growth factors at the top layer of the centrifuged tube simultaneously during harvesting fat by a laboratory assistant and simultaneously during surgery at the operation room. Peripheral blood (10 mL) was collected from each patient into an Acti-PRP tube (Aeon Biotherapeutics Corp., Taipei, Taiwan). The tubes were centrifuged at 300 rpm for 6 min. The plasma and buffy coat in the upper layer above the separation gel were harvested and thoroughly mixed. The Acti-PRP tubes were allowed to remain on the test tube rack for 20 min until the platelet-rich fibrin (PRF) formed. The tubes with PRF were centrifuged 1 or 2 times until the PRF was completely compressed to a thin layer above the separation gel, thus releasing the PRP [12]. Then 2 mL of the PRP was collected by a syringe at room temperature and prepared to be mixed with fat for injection.

The harvested emulsified fat was then mixed with PRP in a volume ratio of 4:1. Then, the fat alone or the fat–PRP mixture was drawn into a Brüning’s syringe prepared for injection laryngoplasty. The suspension laryngoscopy was then performed by a senior laryngologist with a dental protector. The laryngoscopy was fixed, and the microscope was turned on and focused, and the vocal folds were visualized on the monitor. The fat or fat–PRP mixture (1.5 mL) was then injected into the midcord and postcord between 1/3 and 1/4 of the vocal fold width at the free edge lateral side of the thyroarytenoid (TA) muscle to cause bulking of the atrophic vocal fold.

### 2.2. Assessments

#### 2.2.1. Videostroboscopic Examination

The condition of the sulcus was assessed using a laryngeal stroboscopy (KayPentax, Model #RLS 9100-B, Montvale, NJ, USA). The findings were analyzed and sorted into three categories (disappeared, improved, or no change) by comparing the size of the vocal gap at the follow-up visits with that before the procedure. Videostroboscopy was used to observe supraglottic constriction, which was scored using a stroboscopy examination rating form (SERF). The distances of the front to rear and left to right of the glottis were measured. A 5-point scoring system was used to grade a vocal cycle in a montage photo (contained 10 figures in a closed phase of a vocal cycle) by stroboscopy montage program: a score of 0, normally closed (5 closed and 5 mild glottal gap); a score of 1, slightly toward opened phase (4 closed and 6 mild glottal gap); a score of 2, medium toward open phase (3 closed and 7 mild glottal gap); a score of 3, marked toward opened phase (2 closed and 8 mild glottal gap); a score of 4, severe toward opened phase (1 closed and 9 mild glottal gap) [13]. The glottal gap was graded subjectively by the same experienced speech pathologist to evaluate the anterior–posterior and left–right gaps in this montage image under the SERF grading system as the description of glottal closure condition in all of the sulcus patients.

#### 2.2.2. Acoustic Analysis

A multidimensional voice program (MDVP, Model 4500; Kay Elemetrics Corp., Lincoln Park, NJ, USA) was used to assess voice parameters. Prior to the injections, a voice assessment was conducted by a trained assessor (experienced speech pathologist) in an audiometer testing room, and the room temperature was set at 22 °C [14]. The patients were asked to take a deep breath, and with their mouth 10 cm from the microphone were asked to sustain the vowel “a”. The at least 5 s sound was collected, and the sound parameters, i.e., Jitter, Shimmer, noise to harmonic ratio (NHR), voice turbulence index (VTI), and soft phonation index (SPI) were analyzed, and the maximum phonation time (MPT) was also calculated by a stopwatch during the sustained utterance of the vowel “a” [14]. VTI represented the high-frequency voice noise to measure the voice turbulence and was calculated as the ratio of high-frequency noise to total energy [15]. SPI is the ratio of low frequency (from 70 to 1600 Hz) to higher frequency (from 1600 to 4500 Hz) in harmonic energy and used to assess the weakness of high-frequency harmonic components [16].

#### 2.2.3. Grade, Roughness, Breathiness, Asthenia, and Strain Scale (GRBAS)

GRBAS is a clinician-based acoustic assessment protocol used by a speech pathologist and not the surgeon to assess the speech quality of a sustained utterance of the vowel “a” before and after surgery in a soundproof room at the voice center [17]. It was used for the perceptual voice analysis by a speech pathologist before and after surgery. The GRBAS score was judged on a scale of 0–3 (a score of 0, normal; a score of 1, mild; a score of 2, moderate; and a score of 3, severe hoarse voice).

#### 2.2.4. Voice Handicap Index

VHI-10 is a questionnaire used to assess the degree of voice handicap. It contains 10 questions that patients answer regarding how frequently the statements about the effects of their voice affect their lives [18]. Each question is graded from 0 to 4, and the final score ranges from 0 to 40. VHI-10 is a concise tool for the initial and follow-up assessment of voice disorders [19] and has excellent internal consistency and reliability (Cronbach alpha = 0.97; intraclass correlation coefficient (ICC) = 0.99) [20]. Some studies also proved that VHI-10 is a valid and sensitive assessment tool for voice disorders [21,22].

### 2.3. Statistical Analysis

The statistical analysis was performed using SPSS version 25.0 software (SPSS Inc., Chicago, IL, USA). Descriptive statistics were presented as mean ± standard deviation. The Kolmogorov–Smirnov test was used to assess the normality of data, and all assessed data were normally distributed (*p* > 0.05). The t-test was used to compare differences in patients’ demographic data between autologous fat injection and autologous fat plus PRP groups. Category variables were presented as counts and percentages and compared between 2 groups by the chi-squared test. The preoperative values and those at 1 month and 6 months postoperatively (3 times) of the videostroboscopic examination, acoustic analysis, GRBAS score, and voice handicap index were compared within and between the groups (2 groups). The two-way repeated measures ANOVA (2 groups × 3 times) were used to analyze the outcomes and followed by a Bonferroni post hoc test. Since different acoustic characteristics measured by spectrogram exist between males and females, we also wanted to survey if gender affects the voice outcome improvement before and after surgery (autologous fat injection vs. autologous fat plus PRP). The nonparametric analysis (Mann–Whitney U test) was used to compare the differences in the acoustic characteristic after the surgeries by gender. A value of *p* < 0.05 was considered to indicate statistical significance.

## 3. Results

A total of 34 patients with type II sulcus vocalis were included in the study, and 17 received an injection of fat alone, and 17 of them received an injection of fat plus PRP (Table 2). All patients completed the study and follow-up, and there were no adverse events. The two groups were comparable with respect to age, weight, height, sex, and the side of vocal atrophy (all, *p* > 0.05).

Changes in VHI-10, GRBAS, and stroboscopy examination were compared between the two groups, and the results are summarized in Table 3.

### 3.1. Outcomes of Fat Alone Group versus Fat Plus PRP Group

Calculating the composite score of AHI-10 and GRBAS, the main effects of group (F = 1.50, *p* = 0.23, η^2^ = 0.08; F = 0.76, *p* = 0.39, and η^2^ = 0.04, respectively), time (F = 52.64, *p* = 0.01, η^2^ = 0.87; F = 52.04, *p* = 0.01, and η^2^ = 0.86, respectively), and time × group (F = 5.89, *p* = 0.01, η^2^ = 0.44; F = 2.83, *p* = 0.09, and η^2^ = 0.27, respectively) were observed. For anterior–posterior and left–right of stroboscopy examination, the ANOVA results revealed the main effects of group (F = 2.11, *p* = 0.16, η^2^ = 0.11; F = 3.28, *p* = 0.08, and η^2^ = 0.17, respectively), time (F = 90.26, *p* = 0.01, η^2^ = 0.92; F = 79.70, *p* = 0.01, and η^2^ = 0.91, respectively), and time × group (F = 2.53, *p* = 0.11, η^2^ = 0.25; F = 1.76, *p* = 0.21, and η^2^ = 0.19, respectively). Post hoc tests indicated the preoperative VHI-10, GRBAS, and stroboscopy examination results were similar between the two groups (all, *p* > 0.05). In both groups, there were significant decreases in the VHI-10, total GRBAS score, and the individual GRBAS items of grade and roughness between the preoperative values and those at 1 month and 6 months postoperatively (all, *p* < 0.05). With respect to VHI-10, at 1 month, the postoperative score of autologous fat plus PRP group was significantly lower than that of the autologous fat-only group (*p* = 0.04). Stroboscopy examinations showed that the anterior–posterior and left–right supraglottic constrictions were significantly decreased in both groups at 1 month postoperatively (both, *p* < 0.05), and there were significant differences between the two groups at 6 months postoperatively. As compared to the baseline, the anterior–posterior and left–right supraglottic constriction values were significantly lower in the autologous fat plus PRP group (*p* = 0.001, *p* = 0.001, respectively) at 6 months postoperatively.

The voice outcomes were represented in the ANOVA table (Table 4). The post hoc tests revealed no differences in acoustic analysis variables between the two groups preoperatively (all, *p* > 0.05, Table 5). At 1 month postoperatively, the MPT was significantly improved in the autologous fat injection group and autologous fat plus PRP group (*p* = 0.04, *p* = 0.04, respectively). At 6 months postoperatively, significant improvement was noted in the autologous fat plus PRP group (*p* < 0.05), but not in the fat-only group. Significant decreases in NHR, Jitter, and Shimmer in both groups were noted at 6 months postoperatively (all, *p* < 0.05). However, the values of these variables in the autologous fat plus PRP group were significantly better than those in the autologous fat-only group at 1 month and 6 months postoperatively (all, *p* < 0.05). There were no different changes before and after surgery 1 month and 6 months for the VTI and SPI.

### 3.2. Acoustic Characteristic of Fat-Only Group versus Fat Plus PRP Group by Gender

Among all the enrolled type II sulcus vocalis patients, the autologous fat injection group (7 male and 10 female patients) was compared with the autologous fat plus PRP group (8 male and 9 female patients). The acoustic characteristic, i.e., high frequency, low frequency, NHR, Jitter, and Shimmer were compared in males and females, respectively. In the male patients (Table 6), there were no significant differences in high frequency and low frequency between the two groups at 1 month and 6 months postoperatively (*p* > 0.05). Compared to the autologous fat injection, the NHR and Jitter were significantly decreased at 1 month postoperatively in the autologous fat plus PRP group (*p* = 0.01 and 0.04, respectively), and the decrease in NHR was significant at 6 months postoperatively (*p* = 0.001). In the female patients (Table 7), no significant differences in high frequency and low frequency were also noted between the two groups at 1 month and 6 months postoperatively (*p* > 0.05). We found the patients in the autologous fat plus PRP group had significantly decreased in Shimmer compared with those in the autologous fat injection group (*p* = 0.04). The decreases in NHR, Jitter, and Shimmer were also significantly found in the autologous fat plus PRP group at 6 months postoperatively (*p* = 0.001, 0.04, and 0.01, respectively). In all of the patients, significant decreases in the NHR, Jitter, and Shimmer in both groups were noted at 1 month and 6 months postoperatively (all, *p* < 0.05). These results were similar to the results in male or female patients. Hence, the improvements in voice quality in autologous fat plus PRP were found in male, female, and all patients. Although there were different acoustic characteristics between males and females, but concerning the before and after surgery (autologous fat injection vs. autologous fat plus PRP), we did not find the gender differences in acoustic characteristics in the current study.

## 4. Discussion

This is the first study to use a comprehensive evaluation, including videostroboscopic examination, acoustic analysis, GRBAS, and VHI-10 to assess the outcomes of injection laryngoplasty in patients with vocal atrophy and sulcus vocalis type II. Severities of atrophy of the SLP will cause different vocal sulcus and conditions of dysphonia. The type of sulcus vocalis is difficult to confirm by a laryngoscope or stroboscope alone. Recent reports have indicated that digital imaging with laryngeal stroboscopy can improve the accurate diagnostic rate for sulcus vocalis [23,24,25]. A videostroboscopic examination can provide detailed information regarding the degree of atrophy of the SLP, and thus assist in making a more accurate diagnosis of the type of sulcus vocalis. Meanwhile, it has been reported that the severity of the vocal sulcus is positively correlated with voice quality [23]. Poorer voice quality is found in type II sulcus vocalis when compared to type I sulcus vocalis. Therefore, an accurate diagnosis of the type of vocal sulcus requires MDVP-measured Jitter, Shimmer, and noise to harmonic ratio. In the current study, the measures of front to rear and left to right glottis distances and voice quality were assessed by laryngeal stroboscopy and acoustic assessment, respectively. The improvements noted by stroboscopy in patients after injections were consistent with the results of acoustic assessment showing increases in voice quality and decreases in voice handicap.

Despite no differences in GRBAS and MPT between the fat plus PRP and the fat-only group 1 month and 6 months after injection laryngoplasty, the fat plus PRP group had significantly less Jitter and Shimmer measured by MDVP 1 month and 6 months after injection than the fat-only group did. Softened vocal folds were also noted in the fat plus PRP group under a postoperative stroboscopic survey; less variability in the signal was noted in the fat plus PRP group. Better vocal vibration and recovery of the mucosa waves and sustained less variability in the signal 1 month and 6 months after injection were found in the fat plus PRP group. However, previous studies have indicated that short-term outcomes were the same as those with an injection of autologous fat only or PRP only [26]. The mixture of fat and PRP, which was less condensed when compared to the fat-only group might contribute to better pliable vocal movement. This might explain why less variability in the signal in Jitter, Shimmer, and NHR was found in the fat plus PRP group at the early stage of vocal fold recovery.

We found no VTI or SPI changes before and after injection laryngoplasty in both groups. VTI was calculated by the Multi-Dimensional Voice Program of CSL (computerized speech lab) 4500 from Kay Elemetrics as the ratio of high-frequency noise to total energy and represented the high-frequency voice noise to measure the voice turbulence caused by the vocal sulcus-related incomplete vocal fold adduction. Thus, injection laryngoplasty by fat mixed with PRP or not had no significant effect on high-frequency voice noise. SPI was also measured by utilizing the Multi-Dimensional Voice Program in CSL 4500, which signified the approximation of vocal folds. The fact that no SPI or VTI differences were found between both groups means there were no significant approximation differences between these two groups. The vocal scaring associated with sulcus vocalis resulted in worse voice quality. Voice therapy can improve voice quality, but the overall outcomes are not predictable. Several nonsurgical and surgical methods can treat sulcus vocalis. Voice therapy is more effective for less severe sulcus vocalis, such as type I, whereas more severe sulcus vocalis, such as type II, is less responsive to voice therapy [4].

Sulcus vocalis can be treated by CO_2_ laser, and transverse resection with the application of tissue regeneration material can improve voice quality and a patient’s quality of life [2]. A prior study examined laryngeal coblation to release vocal sulcus scarring where the epithelium was almost completely adhered to the vocal ligament due to a total loss of the SLP, followed by the application of autologous fat, tissue growth factor, or PRP to the coblated wound [27]. The results showed an improvement in voice quality, but the sample size was small. Minithyroidotomy is a less invasive surgery in which the vocal epithelium is dissected from the vocal ligament. Then autologous fat is infused, thus preserving the vocal fold without cutting or injuring the vocal mucosa [28]. The study results showed an improvement in voice quality, but the number of patients was small.

Microflap development and fat transplant with vocal suture was described by Sataloff et al. [29] and was associated with partial recovery of dysphonia. However, it is sometimes difficult to develop a microflap because of the severely adhered epithelium. Fat leakage after suturing the flap is also a cause of unpredictable outcomes. In addition, suturing the microflap is time consuming and requires a high level of surgical expertise. Some authors have described microflap followed by a transplant of autologous temporalis fascia to prevent the recurrence of the vocal epithelium adhering to the vocal ligament, migration of the fascia, or suture loss due to a postoperative laryngeal irritation cough; however, these factors cause the procedure to have unpredictable outcomes. Pontes and Behlau reported a method in which a Z-plasty is performed on the sulcus vocalis [30].

Various pulse dye laser methods, photodynamic therapy, and cryotherapy have been examined to treat sulcus vocalis, but these methods are not commonly used because of potential injury to the vocal folds and further vocal scarring [24]. Injection laryngoplasty is considered a less aggressive method for treating sulcus vocalis. Materials injected include steroids, HA, mesenchymal stem cells, autologous fibroblasts, adipose-derived stem cells, hepatocyte growth factor, basic fibroblast growth factor, pirfenidone, and other anti-fibrotic agents [31]. Other materials used include autologous fat, PRP, platelet lysate, Alloderm, artificial collagen, and Atelocollagen [32]. Fat injection can be considered space occupying and regenerative. Fat remains in place longer than other materials do. Because of the possibility that fat-derived stem cells are harvested during fat harvesting, some authors have postulated that fat injection holds promise as a curative treatment for diseased vocal folds [33]. Autologous fat transfer is used in various plastic surgery procedures, such as mammoplasty and facial lifting procedures, suggesting that fat transfer could also be used to treat vocal defects of various etiologies [34].

There was sustainably better Jitter, Shimmer, and NHR in the fat plus PRP group than the fat group at 1 month and 6 months after injection. We also found more significant differences in gap distance between the fat-only group and the fat plus PRP group measured by stroboscopy postoperatively. Therefore, we presume that the fat reabsorption was greater in the fat-only group than the fat plus PRP mixture group. The fat absorption rate ranges from 40% to 65% per year [35]. Some studies have revealed that certain materials can prolong fat absorption. Basic fibroblast growth factor (bFGF) and various growth factors could be used to prolong the survival duration of autologous fat [36]. The lack of growth factors might accelerate the absorption of injected fat in the vocalis area. Collecting all these growth factors is difficult and not economical. PRP contains high concentrations of growth factors, such as bFGF, hepatocyte growth factor (HGF), and epidermal growth factor (EGF), as well as various cytokines that promote tissue regeneration [37]. Thus, we chose a platelet-derived material to slow the absorption of the injected fat for PRP contains abundant growth factors. We assumed that fat is absorbed faster in the fat-only group, which explained why there were no significant improvements in MPT and noticeable gaps in stroboscopic findings in the 6-month follow up. However, the fat-only group still rendered a significantly decreased Jitter, Shimmer, and NHR at 6 months after injection. On the other hand, fat remained longer in the fat plus PRP group. Sustained better improvements in measures of voice quality by Jitter, Shimmer, and NHR were found in the fat plus PRP group when compared to fat-only group 6 months after injection.

Autologous PRP has been widely used in various kind of diseases [38,39,40,41,42], for it contains abundant growth factors simulating proliferation and repairing of cells. It can be applied to the patients with degenerative joint disease of the knee, shoulder, spine, and other joints or myofascial pain and chronic pain as a result of fascia, ligament, and muscle degeneration [37]. PRP has also been proven to help to stabilize the implantation of the fertilized ovum [43]. PRP contains high concentrations of growth factors, such as bFGF, hepatocyte growth factor (HGF), and epidermal growth factor (EGF), as well as various cytokines that promote tissue regeneration [37]. It also contains platelet-releasing growth factors and inflammatory cytokines associated with platelets [37]. A study showed that PRP enhanced fat survival fourfold when the fat was transferred to fill skin defects [44]. In an animal study, PRP was shown to accelerate the recovery of injured rat vocal folds. The treatment was associated with less collagen, fewer fibroblasts, and a greater level of HA [45]. The results also showed that PRP treatment was associated with less infiltration of lymphocytes, higher viability, and better integration of fatty cells [45]. Because a high concentration of growth factors could naturally activate adipose cells to grow, we could also adjust the number of growth factors according to various injured or atrophic vocal conditions. In addition to high levels of growth factors, the depletion of WBC in PRP prevents tissue inflammation because of a reduced immune response due to WBC [46]. Therefore, the mixture of autologous fat and PRP renders more prolonged fat survival and less inflammation of the vocal folds, resulting in better and more prolonged voice outcomes. More studies are needed to determine if the fat plus PRP mixture can promote the vocalis muscle’s regeneration and render prolonged, stable voice outcomes.

Autologous fat injection laryngoplasty promotes vocalis rejuvenation and better vocal edge contouring and is considered a regenerative surgery for glottic insufficiency. However, the fat itself is still not stable, and the quality may differ in each patient. Patients who are older and thinner usually have poor fat quality and less fat to harvest. As such, PRP can help the fat that is available survive longer [47,48]. Studies have shown that PRF and PRP can improve the outcomes of fat grafting for tissue defects [49,50]. However, PRP contains higher levels of anti-inflammatory factors and growth factors than PRF. When PRP is produced, anti-inflammatory cytokines and growth factors are nearly totally released from the platelets. The WBC is almost wholly removed; thus, the anti-inflammatory effect is better with PRP [50]. The PRP may enhance fat and nanofat survival as evidenced in a mouse model [51]. PRP has also been shown to assist in fat distribution in the defect after fat grafting and thus improve the distribution of fat in the vocalis muscle resulting in asymmetric vocal fold movement and better vocal vibration [52]. In addition, it would be important to note that dysphonia from the sulcus is not only due to glottal insufficiency alone but also due to disrupted vibration due to increased cover layer stiffness. Thus, improving the glottal gap by fat alone will not fully treat the dysphonia. It is possible that PRP prevented fat from diffusion in the sulcus after injection and even had the antifibrotic effects, whereas the fat alone cases were medializing alone.

The use of the PRP plus fat mixture is associated with faster wound healing and a shorter recovery time than when fat alone is used [53]. PRP promotes angiogenesis and adipogenesis, inhibits apoptosis, and regulates collagen production, resulting in improved fat distribution and survival [54,55]. Microscopically, the area surrounding the injected fat is well vascularized, contributing to the improvement of the vocalis muscle [56]. An injection of fat plus PRP results in a longer duration of filling the tissue defect and increased volume and softening of the tissue [57]. The fat plus PRP mixture also prevents fibrosis in post-surgical tissue and, therefore, may also prevent post-LMS vocal fibrosis. In fact, the mixture can be injected underneath the dissected sulcus area to avoid the re-adhesion of the vocal epithelium to the vocal ligament [58].

The voice quality recovered rapidly, as demonstrated in self-perceived VHI-10 by the patients with sulcus. In addition, the objective evaluation by MDVP also showed less Jitter and Shimmer in the fat plus PRP mixture group than the fat-only group. There have been reports of increased vocal stiffness after autologous fat injection laryngoplasty, as well as reports of laryngeal infection. Inflammation occurs when fat is injected into the vocalis muscle area, and the addition of PRP can prevent this. Thus, less inflammation and less fibrosis of the vocal folds lead to better recovery of the mucosa wave, better voice quality, and quicker recovery from vocal strain. In addition, we also surveyed whether gender affects the outcomes by autologous fat injection versus autologous fat plus PRP. We finally found the similar voice outcome improvement in males and females. Table 6 and Table 7 show that there were no gender differences in the improvement between male and female groups.

## 5. Conclusions

In summary, fat plus PRP injection leads to more remarkable softening of the vocal folds, and better vibration, and better mucosa waves lead to better voice quality. Fat plus PRP is associated with a sustained better function, as evidenced by a longer MPT and lower GRBAS total score. Our results show that the fat plus PRP mixture is an effective alternative to fat-only injection laryngoplasty and is associated with longer improved voice quality because of more prolonged fat survival due to the PRP. We thought that Fat plus PRP injection laryngoplasty is a promising method for treating type II sulcus vocalis. However, the limitations of this study include the small number of patients and the different sulcus areas in the vocal folds, and the etiology may remain unresolved.

## Figures and Tables

**Table 1:** Inclusion and exclusion criteria in the current study.

Inclusion Criteria	Exclusion Criteria
Patients with dysphonia with stroboscopy and laryngomicroscopy revealed type II sulcus. Patients with no systemic blood disorders and able to tolerate laryngomicroscopic surgery under general anesthesia. Dysphonia because of vocal sulcus and voice quality not recovered by voice therapy. Patients accept phonosurgery to treat sulcus-related dysphonia.	Patients with bleeding tendency. Underlying disease needs anticoagulants, which could not be ceased for their general condition, not allowed to stop anticoagulants. Vocal palsy-related dysphonia. Spasmodic dysphonia or neurological disorders, related dysphonia. Organic lesions were found, e.g., vocal polyps (or nodules), vocal tumor, vocal cyst, or glottic cancer. Vocal scarring without sulcus. Patients received phonosurgery before. Patients with high expectation of voice outcome by fat with or without PRP injection laryngoplasty. Patients with psychological disorder.

**Table 2:** Demographic data of the patients.

	Autologous Fat Injection Group (*n* = 17)	Autologous Fat plus PRP Group (*n* = 17)	*p* Value
Age (years)	57.21 ± 10.68	60.12 ± 9.89	0.41
Weight (kg)	75.67 ± 15.78	76.33 ± 18.21	0.91
Height (cm)	171.34 ± 10.56	169.78 ± 11.84	0.68
Sex (no., %)			
Male	7 (41.17%)	8 (47.06%)	0.11
Female	10 (58.82%)	9 (52.95%)	
Side, (no., %)			
Left	4 (23.53%)	3 (17.65%)	0.81
Right	2 (11.76%)	2 (11.76%)	
Bilateral	11 (64.71%)	12 (70.59%)	

**Table 3:** Pre- and postoperative (1 month and 6 months) results in VHI-10, GRBAS, and stroboscopy examination.

	Autologous Fat Injection Group (*n* = 17)			Autologous Fat plus PRP Group (*n* = 17)		
	Preoperative	Postoperative (1 Month)	Postoperative (6 Months)	Preoperative	Postoperative (1 Month)	Postoperative (6 Months)
VHI-10	26.92 ± 7.76 ^c^	21.88 ± 7.36 ^a^	16.69 ± 5.35 ^b^	28.13 ± 10.81 ^c^	17.12 ± 6.41 ^a,d^	12.47 ± 5.21 ^b^
GRBAS						
G	2.54 ± 0.52 ^c^	1.41 ± 0.63 ^a^	0.78 ± 0.63 ^b^	2.01 ± 0.79 ^c^	1.11 ± 0.59 ^a^	0.69 ± 0.42 ^b^
R	2.16 ± 0.76 ^c^	1.32 ± 0.43 ^a^	0.67 ± 0.43 ^b^	1.76 ± 0.83 ^c^	0.83 ± 0.21 ^a^	0.63 ± 0.22 ^b^
B	1.78 ± 0.85	1.55 ± 0.67	0.75 ± 0.98	1.65 ± 1.01	0.79 ± 0.68 ^a^	0.80 ± 0.52
A	1.38 ± 0.97	1.28 ± 0.87	0.66 ± 1.32	1.29 ± 1.10	1.13 ± 0.83	0.98 ± 0.82
S	1.15 ± 0.74	1.14 ± 0.83	1.10 ± 0.92	1.47 ± 0.94	1.09 ± 0.89	1.01 ± 0.61
Total	7.52 ± 2.61 ^c^	5.68 ± 2.45 ^a^	3.38 ± 1.98 ^b^	6.86 ± 3.79 ^c^	4.45 ± 2.53 ^a^	3.11 ± 1.01 ^b^
Stroboscopy examination						
Anterior–posterior	2.29 ± 0.38	1.08 ± 0.45 ^a^	2.02 ± 1.98	2.32 ± 0.42 ^c^	0.98 ± 0.55 ^a^	1.03 ± 0.47 ^d^
Left–right	2.49 ± 0.45	1.06 ± 0.34 ^a^	1.98 ± 1.87	2.44 ± 0.52 ^c^	1.02 ± 0.45 ^a^	1.12 ± 0.32 ^d^

VHI-10, voice handicap index-10. ^a^ *p* < 0.05, preoperative vs. postoperative 1 month; ^b^ *p* < 0.05, postoperative 1 month vs. postoperative 6 months; ^c^ *p* < 0.05, postoperative 6 months vs. preoperative; and ^d^ *p* < 0.05, autologous fat injection vs. autologous fat plus PRP.

**Table 4:** The voice outcome of ANOVA with the factors for the assessed variables.

	Group			Time			Group × Time		
	F	*p*	η^2^	F	*p*	η^2^	F	*p*	η^2^
MPT	0.77	0.39	0.04	17.21	0.02 *	0.69	0.65	0.53	0.08
High frequency (Hz)	0.05	0.90	0.04	2.86	0.09	0.27	0.18	0.83	0.02
Low frequency (Hz)	0.03	0.81	0.01	3.23	0.07	0.30	1.29	0.31	0.14
NHR	10.72	0.01 *	0.40	9.65	0.01 *	0.56	0.39	0.68	0.05
VTI	0.05	0.81	0.02	4.01	0.04 *	0.34	0.69	0.51	0.08
SPI	0.06	0.78	0.01	2.87	0.08	0.26	0.11	0.90	0.01
Jitter (%)	0.81	0.37	0.04	59.85	0.01 *	0.87	7.97	0.01 *	0.51
Shimmer (%)	2.72	0.11	0.14	58.12	0.01 *	0.78	8.05	0.01 *	0.49

* *p* < 0.05; MPT, maximum phonation time; NHR, noise to harmonic ratio; VTI, voice turbulence index; and SPI, soft phonation index.

**Table 5:** Pre-and postoperative (1 month and 6 months) results in acoustic analysis.

	Autologous Fat Injection Group (*n* = 17)			Autologous Fat plus PRP Group (*n* = 17)		
	Preoperative	Postoperative (1 Month)	Postoperative (6 Months)	Preoperative	Postoperative (1 Month)	Postoperative (6 Months)
MPT	6.79 ± 2.21	8.32 ± 2.14 ^a^	8.89 ± 6.98	7.12 ± 2.24 ^c^	8.65 ± 2.02 ^a^	10.76 ± 3.56 ^b^
High frequency (Hz)	236.74 ± 78.32	247.64 ± 81.11	235.73 ± 77.47	240.12 ± 66.89	249.32 ± 92.33	233.43 ± 78.56
Low frequency (Hz)	150.21 ± 55.63	143.13 ± 63.84	138.26 ± 65.35	141.36 ± 69.83	139.98 ± 80.47	140.77 ± 76.88
NHR	0.35 ± 0.32	0.29 ± 0.17	0.20 ± 0.06 ^b^	0.25 ± 0.21 ^c^	0.14 ± 0.16 ^a,d^	0.04 ± 0.01 ^b,d^
VTI	0.12 ± 0.11	0.07 ± 0.08	0.08 ± 0.12	0.09 ± 0.18	0.07 ± 0.13	0.08 ± 0.11
SPI	24.33 ± 13.34	23.65 ± 12.20	22.66 ± 13.09	23.67 ± 12.59	22.45 ± 13.63	21.98 ± 12.56
Jitter (%)	5.82 ± 1.98 ^c^	5.51 ± 2.01	4.05 ± 1.10 ^b^	6.77 ± 1.66 ^c^	4.34 ± 1.38 ^a,d^	3.02 ± 1.61 ^b,d^
Shimmer (%)	12.72 ± 3.12 ^c^	12.45 ± 3.32	10.21 ± 2.67 ^b^	13.86 ± 2.97 ^c^	10.01 ± 3.22 ^a,d^	7.18 ± 2.35 ^b,d^

MPT, maximum phonation time; NHR, noise to harmonic ratio; VTI, voice turbulence index; SPI, soft phonation index. ^a^ *p* < 0.05, preoperative vs. postoperative 1 month; ^b^ *p* < 0.05, postoperative 1 month vs. postoperative 6 months; ^c^ *p* < 0.05, postoperative 6 months vs. preoperative; and ^d^ *p* < 0.05, autologous fat injection vs. autologous fat plus PRP.

**Table 6:** The results of acoustic characteristic in male patients.

	Autologous Fat Injection Male (*n* = 7)			Autologous Fat plus PRP Group Male (*n* = 8)		
	Preoperative	Postoperative (1 Month)	Postoperative (6 Months)	Preoperative	Postoperative (1 Month)	Postoperative (6 Months)
High frequency (Hz)	160.57 ± 44.84	169.29 ± 47.74	170.71 ± 46.14	188.88 ± 37.28	169.63 ± 45.21	168.75 ± 48.83
Low frequency (Hz)	115.43 ± 21.90	108.43 ± 44.58	95.01 ± 32.02	93.13 ± 19.07	87.25 ± 15.04	90.02 ± 14.78
NHR	0.50 ± 0.39	0.35 ± 0.19	0.22 ± 0.07	0.26 ± 0.17	0.09 ± 0.04 ^a^	0.03 ± 0.02 ^b^
Jitter (%)	6.57 ± 1.51	6.29 ± 1.80	4.57 ± 1.13	7.13 ± 1.83	4.38 ± 1.30 ^a^	3.34 ± 2.24
Shimmer (%)	13.02 ± 2.94	12.64 ± 2.21	10.20 ± 2.52	15.13 ± 2.10	11.70 ± 2.21	8.16 ± 2.12

^a^ *p* < 0.05, autologous fat injection male vs. autologous fat plus PRP group male in postoperative (1 month); ^b^ *p* < 0.05, autologous fat injection male vs. autologous fat plus PRP group male in postoperative (6 months).

**Table 7:** The results of acoustic characteristic in female patients.

	Autologous Fat Injection Female (*n* = 10)			Autologous Fat plus PRP Group Female (*n* = 9)		
	Preoperative	Postoperative (1 Month)	Postoperative (6 Months)	Preoperative	Postoperative (1 Month)	Postoperative (6 Months)
High frequency (Hz)	289.70 ± 42.91	301.61 ± 48.75	281.12 ± 60.20	286.56 ± 50.43	319.11 ± 60.62	291.02 ± 48.25
Low frequency (Hz)	174.50 ± 59.87	168.20 ± 64.41	169.51 ± 66.75	184.33 ± 69.53	186.04 ± 87.60	185.89 ± 81.83
NHR	0.25 ± 0.21	0.24 ± 0.16	0.19 ± 0.07	0.25 ± 0.25	0.20 ± 0.21	0.04 ± 0.02 ^b^
Jitter (%)	5.30 ± 2.06	5.04 ± 2.05	3.72 ± 0.95	6.44 ± 1.59	4.26 ± 1.51	2.76 ± 0.98 ^b^
Shimmer (%)	12.52 ± 3.50	12.30 ± 4.04	10.21 ± 3.01	12.78 ± 3.19	8.56 ± 3.40 ^a^	6.33 ± 2.24 ^b^

^a^ *p* < 0.05, autologous fat injection male vs. autologous fat plus PRP group male in postoperative (1 month); ^b^ *p* < 0.05, autologous fat injection male vs. autologous fat plus PRP group male in postoperative (6 months).

## Data Availability

Data is contained within the article.

## References

[1] Ford C.N., Inagi K., Khidr A., Bless D.M., Gilchrist K.W. (1996). Sulcus vocalis: A rational analytical approach to diagnosis and management. Ann. Otol. Rhinol. Laryngol..

[2] Miaśkiewicz B., Szkiełkowska A., Gos E., Panasiewicz A., Włodarczyk E., Skarżyński P.H. (2018). Pathological sulcus vocalis: Treatment approaches and voice outcomes in 36 patients. Eur. Arch. Otorhinolaryngol..

[3] Lindestad P.A., Hertegard S. (1994). Spindle-shaped glottal insufficiency with and without sulcus vo-calis: A retrospective study. Ann. Otol. Rhinol. Laryngol..

[4] Giovanni A., Chanteret C., Lagier A. (2007). Sulcus vocalis: A review. Eur. Arch. Otorhinolaryngol..

[5] Watson G.J., Jones P.H. (2011). Videographic documentation of an open cyst converting into a sulcus vocalis. J. Voice.

[6] Van den Broek E., Heijnen B.J., Hendriksma M., Langeveld A., van Benthem P., Sjögren E.V. (2019). Bilateral trial vocal fold injection with hyaluronic acid in patients with vocal fold atrophy with or without sulcus. Eur. Arch. Otorhinolaryngol..

[7] Lisi C., Hawkshaw M.J., Sataloff R.T. (2013). Viscosity of materials for laryngeal injection: A review of current knowledge and clinical implications. J. Voice.

[8] Shindo M.L., Zaretsky L.S., Rice D.H. (1996). Autologous fat injection for unilateral vocal fold paraly-sis. Ann. Otol. Rhinol. Laryngol..

[9] Özgürsoy S.K., Tunçkaşık F., Tunçkaşık M.E., Akıncıoğlu E., Doğan H., Beriat G.K. (2018). Histo-pathologic evaluation of hyaluronic acid and plasma-rich platelet injection into rabbit vocal cords: An experimental study. Turk. Arch. Otorhinolaryngol..

[10] Sakalys D., Rokicki J.P., Januzis G., Kubilius R. (2020). Plasma rich in growth factors injection effec-tiveness for myofascial pain treatment in masticatory muscles. Randomised controlled trial. J. Oral Rehabil..

[11] Chang W.D., Chen S.H., Tsai M.H., Tsou Y.A. (2021). Autologous fat injection laryngoplasty for unilateral vocal fold paralysis. J. Clin. Med..

[12] Stavrakas M., Karkos P.D., Markou K., Grigoriadis N. (2016). Platelet-rich plasma in otolaryngology. J. Laryngol. Otol..

[13] Poburka B.J., Patel R.R., Bless D.M. (2017). Voice-vibratory assessment with laryngeal imaging (VALI) form: Reliability of rating stroboscopy and high-speed videoendoscopy. J. Voice.

[14] Patel R.R., Awan S.N., Barkmeier-Kraemer J., Courey M., Deliyski D., Eadie T., Paul D., Švec J.G., Hillman R. (2018). Recommended protocols for instrumental assessment of voice: American speech-language-hearing association expert panel to develop a protocol for instrumental assessment of vocal function. Am. J. Speech Lang. Pathol..

[15] Franca M.C. (2012). Acoustic comparison of vowel sounds among adult females. J. Voice.

[16] Akil F., Yollu U., Ozturk O., Yener M. (2017). Differences of the voice parameters between the population of different hearing tresholds: Findings by using the multi-dimensional voice program. Clin. Exp. Otorhinolaryngol..

[17] Shin Y.S., Chang J.W., Yang S.M., Wu H.W., Cho M.H., Kim C.H. (2013). Persistent dysphonia after laryngomicrosurgery for benign vocal fold disease. Clin. Exp. Otorhinolaryngol..

[18] Lam P.K., Chan K.M., Ho W.K., Kwong E., Yiu E.M., Wei W.I. (2006). Cross-cultural adaptation and validation of the Chinese Voice Handicap Index-10. Laryngoscope.

[19] Behlau M., Madazio G., Moreti F., Oliveira G., dos Santos L.d.M.A., Paulinelli B.R., Couto Junior Ede B. (2016). Efficiency and cutoff values of self-assessment instruments on the impact of a voice problem. J. Voice.

[20] Rosen C.A., Lee A.S., Osborne J., Zullo T., Murry T. (2004). Development and validation of the voice handicap index-10. Laryngoscope.

[21] Tafiadis D., Helidoni M.E., Chronopoulos S.K., Kosma E.I., Alexandropoulou A., Velegrakis S., Konitsiotis S., Ziavra N. (2020). ROC analysis cut-off points of hellenic voice handicap index for neurogenic voice disorders patients: An exploratory study. J. Voice.

[22] Tafiadis D., Helidoni M.E., Chronopoulos S.K., Kosma E.I., Ziavra N., Velegrakis G.A. (2020). Cross-cultural adaptation and validation of the Greek voice handicap index-10 (GVHI-10) with additional receiver operating characteristic analysis. J. Voice.

[23] Lim J.Y., Kim J., Choi S.H., Kim K.M., Kim Y.H., Kim H.S., Choi H.S. (2009). Sulcus configura-tions of vocal folds during phonation. Acta Otolaryngol..

[24] Soni R.S., Dailey S.H. (2019). Sulcus Vocalis. Otolaryngol. Clin. N. Am..

[25] Woo P. (2016). 4K video-laryngoscopy and video—Stroboscopy: Preliminary findings. Ann. Otol. Rhinol. Laryngol..

[26] Woo P., Murry T. (2021). Short-term voice improvement after repeated office-based platelet-rich plasma PRP injection in patients with vocal fold scar, sulcus, and atrophy. J. Voice.

[27] Hansen J.K., Thibeault S.L. (2006). Current understanding and review of the literature: Vocal fold scarring. J. Voice.

[28] Mallur P.S., Gartner-Schmidt J., Rosen C.A. (2012). Voice outcomes following the gray minithyrotomy. Ann. Otol. Rhinol. Laryngol..

[29] Sataloff R.T., Spiegel J.R., Hawkshaw M., Rosen D.C., Heuer R.J. (1997). Autologous fat implanta-tion for vocal fold scar: A preliminary report. J. Voice.

[30] Pontes P., Behlau M. (1993). Treatment of sulcus vocalis: Auditory perceptual and acoustical analysis of the slicing mucosa surgical technique. J. Voice.

[31] Courey M.S. (2004). Injection laryngoplasty. Otolaryngol. Clin. N. Am..

[32] Tamura E., Fukuda H., Tabata Y. (2008). Intracordal injection technique: Materials and injection site. Tokai. J. Exp. Clin. Med..

[33] Lasso J.M., Poletti D., Scola B., Gómez-Vilda P., García-Martín A.I., Fernández-Santos M.E. (2018). Injection laryngoplasty using autologous fat enriched with adipose-derived regenerative stem cells: A safe therapeutic option for the functional reconstruction of the glottal gap after unilateral vocal fold paralysis. Stem Cells Int..

[34] Butterwick K.J., Nootheti P.K., Hsu J.W., Goldman M.P. (2007). Autologous fat transfer: An in-depth look at varying concepts and techniques. Facial Plast. Surg. Clin. N. Am..

[35] Kruschewsky L.d.S., de Mello-Filho F.V., dos Santos A.C., Rosen C.A. (2007). Autologous fat graft absorption in unilateral paralyzed canine vocal folds. Laryngoscope.

[36] Tamura E., Tabata Y., Yamada C., Okada S., Iida M. (2015). Autologous fat augmentation of the vocal fold with basic fibroblast growth factor: Computed tomographic assessment of fat tissue survival after augmentation. Acta. Otolaryngol..

[37] Knop E., Paula L.E., Fuller R. (2016). Platelet-rich plasma for osteoarthritis treatment. Rev. Bras. Reumatol. Engl. Ed..

[38] Xu J., Gou L., Zhang P., Li H., Qiu S. (2020). Platelet-rich plasma and regenerative dentistry. Aust. Dent. J..

[39] Peng G.L. (2019). Platelet-rich plasma for skin rejuvenation: Facts, fiction, and pearls for practice. Facial Plast. Surg. Clin. N. Am..

[40] Elghblawi E. (2018). Platelet-rich plasma, the ultimate secret for youthful skin elixir and hair growth triggering. J. Cosmet. Dermatol..

[41] Filardo G., Di Matteo B., Kon E., Merli G., Marcacci M. (2018). Platelet-rich plasma in tendon-related disorders: Results and indications. Knee Surg. Sports Traumatol. Arthrosc..

[42] Anitua E., Fernández-de-Retana S., Alkhraisat M.H. (2021). Platelet rich plasma in oral and maxillofacial surgery from the perspective of composition. Platelets.

[43] Tandulwadkar S.R., Naralkar M.V., Surana A.D., Selvakarthick M., Kharat A.H. (2017). Autologous intrauterine platelet-rich plasma instillation for suboptimal endometrium in frozen embryo transfer cycles: A Pilot Study. J. Hum. Reprod. Sci..

[44] Houdek M.T., Wyles C.C., Stalboerger P.G., Terzic A., Behfar A., Moran S.L. (2016). Collagen and fractionated platelet-rich plasma scaffold for dermal regeneration. Plast. Reconstr. Surg..

[45] Cobden S.B., Oztürk K., Duman S., Esen H., Aktan T.M., Avunduk M.C., Elsurer C. (2016). Treatment of acute vocal fold injury with platelet-rich plasma. J. Voice.

[46] Seria E., Galea G., Borg J., Schembri K., Grech G., Tagliaferro S.S., Felice A. (2021). Novel leukocyte-depleted platelet-rich plasma-based skin equivalent as an in vitro model of chronic wounds: A preliminary study. BMC Mol. Cell Biol..

[47] Smith O.J., Jell G., Mosahebi A. (2019). The use of fat grafting and platelet-rich plasma for wound healing: A review of the current evidence. Int. Wound J..

[48] Xiong S., Yi C., Pu L.L.Q. (2020). An overview of principles and new techniques for facial fat grafting. Clin. Plast Surg..

[49] Xiong S., Qiu L., Su Y., Zheng H., Yi C. (2019). Platelet-rich plasma and platelet-rich fibrin enhance the outcomes of fat grafting: A comparative study. Plast. Reconstr. Surg..

[50] Zamani M., Yaghoubi Y., Movassaghpour A., Shakouri K., Mehdizadeh A., Pishgahi A., Yousefi M. (2019). Novel therapeutic approaches in utilizing platelet lysate in regenerative medicine: Are we ready for clinical use?. J. Cell Physiol..

[51] Lei X., Liu H., Pang M., Zheng Z., Tan X., Cheng B. (2019). Effects of platelet-rich plasma on fat and nanofat survival: An experimental study on mice. Aesthet. Plast. Surg..

[52] Han S., Gan D., Wang G., Ru Y., Huang C., Lin J., Zhang L., Meng Z., Zhu S. (2018). Associations of platelet indices with body fat mass and fat distribution. Obesity.

[53] Smith O.J., Leigh R., Kanapathy M., Macneal P., Jell G., Hachach-Haram N., Mann H., Mosahebi A. (2020). Fat grafting and platelet-rich plasma for the treatment of diabetic foot ulcers: A feasibility-randomised controlled trial. Int. Wound J..

[54] O’Connell B., Wragg N.M., Wilson S.L. (2019). The use of PRP injections in the management of knee osteoarthritis. Cell Tissue Res..

[55] Kobayashi Y., Saita Y., Takaku T., Yokomizo T., Nishio H., Ikeda H., Takazawa Y., Nagao M., Kaneko K., Komatsu N. (2020). Platelet-rich plasma (PRP) accelerates murine patellar tendon healing through enhancement of angiogenesis and collagen synthesis. J. Exp. Orthop..

[56] Yu P., Zhai Z., Lu H., Jin X., Yang X., Qi Z. (2020). Platelet-rich fibrin improves fat graft survival possibly by promoting angiogenesis and adipogenesis, inhibiting apoptosis, and regulat-ing collagen production. Aesthet. Surg. J..

[57] Kim S.H., Park E.S., Kim T.H. (2017). Rejuvenation using platelet-rich plasma and lipofilling for vaginal atrophy and lichen sclerosus. J. Menopausal Med..

[58] Guler S., Akcali O., Sen B., Micili S.C., Sanli N.K., Cankaya D. (2020). Effect of platelet-rich plasma, fat pad and dural matrix in preventing epidural fibrosis. Acta. Ortop. Bras..

